# The Graduates of Our Medical Schools

**Published:** 1857-05

**Authors:** 


*The Graduates of our Medical Schools.*—Such is the title of an editorial article in the Boston Medical Journal of April 23d. It inquires "how many of these graduates have purchased the *prestige* which belongs only to a regular physician?—the sellers, the faculty of the college, knowing full well that the recipient of the degree will in the next month put himself in opposition to all of our noble calling as a homœopathist."

This is a serious question, and one which we confess somewhat surprizes us. We know very well that the Boston *Journal* is not hasty in its statements and not given to indiscriminate attacks on persons or things, merely for the amusement of the fight. We take it for granted that this must have some application, and that a local one, a supposition confirmed by what we have previously known of the conduct of the Massachusetts State Medical Society, in holding within its fellowship numbers of homœopathists; refusing, out of a womanish tenderness, to expel them. Now we do not dislike a man because he is a homœopath, and we have no doubt that many of this class who retain their membership in that venerable society, are gentlemen and clever fellows. We would not hurt their feelings, but we would not permit them to remain as members of a society—medical in its character—to which we belong. One of two things is certain: either the legitimists or the homœopaths are egregiously in the wrong—their doctrines are as widely separated as doctrines can be—and they ought not, in self-respect, to associate with each other professionally. We once met a homœopath at the bedside of a patient of our own, to which he had been unwittingly called. As soon as he discovered the facts there was no difference of opinion—we agreed charmingly. We refused to consult with him, and he, like a gentleman, refused to consult with us; neither could do it without a sacrifice of his own dignity, and we left the house and the patient together like brothers. Now we would take the same ground with reference to a medical society — there is no good end to be gained by this promiscuous intercourse. Were we a homœopath, we would not belong to the Massachusetts Medical Society, and were we a member of that body we would insist on putting the homœopaths out — or else leave ourselves.

But we have wandered — we usually do. The question is, shall a medical school graduate a young man, no matter how able or accomplished, who proposes to practice homœopathy? In the Buffalo school that question has been decided, once for all, *nemine contradicente.* A young man of excellent character and very fair acquirements, was a candidate for a degree. He had studied his office term with a homœopathist; and on that ground he was rejected. The question was not raised what he would do, it was enough that he had not studied three years in the office of a regular practitioner of medicine. It is always better to decide on the lesser question of past history, than on the uncertain enigma of future conduct, and this was the ground taken in this case — but the fear was that he would practice homœopathy, and that would have rejected him had not the unsatisfactory mode of his study come in to interfere.

